# Recent progress in Pickering emulsions stabilised by bioderived particles

**DOI:** 10.1039/d1ra08086e

**Published:** 2021-12-07

**Authors:** Kazi M. Zakir Hossain, Laura Deeming, Karen J. Edler

**Affiliations:** Department of Chemistry, University of Bath Claverton Down Bath BA2 7AY UK K.Edler@bath.ac.uk; Centre for Sustainable Chemical Technologies, University of Bath Claverton Down Bath BA2 7AY UK

## Abstract

In recent years, the demand for non-surfactant based Pickering emulsions in many industrial applications has grown significantly because of the option to select biodegradable and sustainable materials with low toxicity as emulsion stabilisers. Usually, emulsions are a dispersion system, where synthetic surfactants or macromolecules stabilise two immiscible phases (typically water and oil phases) to prevent coalescence. However, synthetic surfactants are not always a suitable choice in some applications, especially in pharmaceuticals, food and cosmetics, due to toxicity and lack of compatibility and biodegradability. Therefore, this review reports recent literature (2018–2021) on the use of comparatively safer biodegradable polysaccharide particles, proteins, lipids and combinations of these species in various Pickering emulsion formulations. Also, an overview of the various tuneable factors associated with the functionalisation or surface modification of these solid particles, that govern the stability of the Pickering emulsions is provided.

## Abstract 2

In a Pickering emulsion, solid particles accumulate at the interface between two immiscible phases to reduce coalescence by forming a physical barrier. Using bioderived particles is becoming popular to generate more sustainable Pickering emulsions.

## Graphical abstract

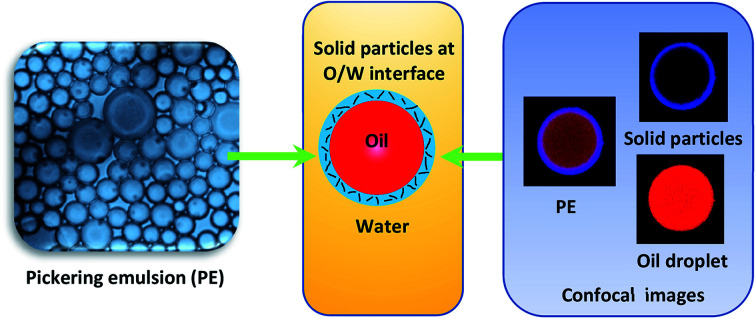

## 1. Introduction

**Fig. 1:**
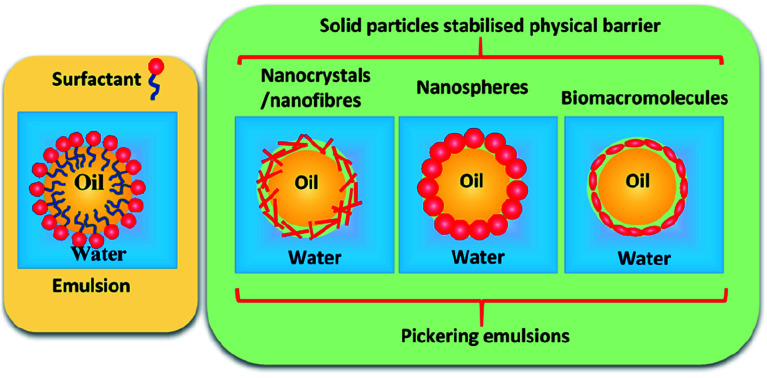
Schematic representation of surfactant stabilised emulsion and solid nanoparticle stabilised Pickering emulsions.

Pickering emulsions are becoming more widely used in many fields, including food, cosmetics, paints, coating, pharmaceutics, and drug delivery,^1–6^ although they were first reported a century ago by Ramsden in 1903 (ref. 7) and Pickering in 1907.^8^ In a Pickering emulsion, solid particles are allowed to accumulate at the interface between two immiscible phases to reduce the possibility of coalescence by forming a physical barrier (see Fig. 1).

A significant variety of inorganic (such as silica, clay) and organic (polysaccharides and proteins) particles have been effectively utilised as Pickering emulsifiers providing long-term emulsion stability.^2–4,9–11^ The key advantages of using solid particles as emulsifiers are associated with their tunable properties (such as wettability, surface charge, porosity, responsiveness *etc.*), and the option to select the particles from sustainable sources with biodegradability and low toxicity.^9^

**Fig. 2:**
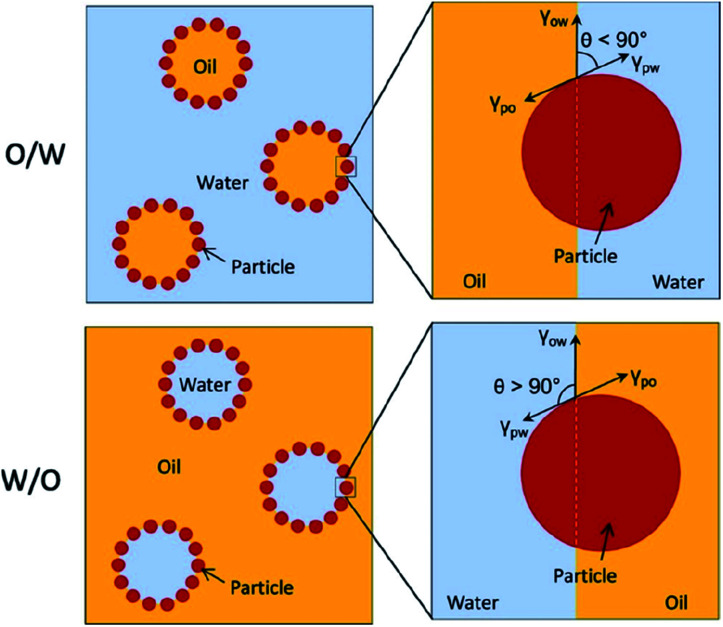
Schematic representation of O/W and W/O-type Pickering emulsions governed by the wettability (contact angle, *θ*) of the solid particles.^4^ Reprinted from ref. 4, Copyright 2019, with permission from Elsevier.

It is very important to understand the mechanism of Pickering emulsion formation when selecting a solid emulsifier. In Pickering emulsions, the solid particles are irreversibly adsorbed at the oil–water interface to form a steric barrier resulting in a more stable emulsion formulation. The mechanism of the attachment of the solid particles at the oil–water interface mainly depends on their wettability, which determines the type of emulsion, either water-in-oil (W/O) or oil-in-water (O/W) systems. Typically, the phase with the better wetting capacity on the solid particles becomes the continuous phase, while the other becomes the dispersed phase. More specifically, in an O/W-type emulsion, the contact angle at the three-phase boundary (*i.e.*, between the disperse phase, solid particles and continuous phase) is less than 90° (*i.e.*, hydrophilic) (see Fig. 2).^4^ A W/O-type emulsion forms if the three-phase contact angle is higher than 90° (usually hydrophobic). However, a more stable Pickering emulsion is formed when the contact angle is close to 90°, where the solid particles balance their dispersion in both phases. Therefore, there is an option for the researchers to alter the surface properties (for example, wettability) of these solid particles to make them more amphiphilic.

Water-in-water (W/W) emulsions can also be formed by dispersing one aqueous phase into another aqueous phase, where the aqueous phases contain two types of hydrophilic macromolecules that are immiscible with each other. The W/W emulsions offer an oil-free system suggesting a more sustainable and greener approach than the traditional O/W or W/O emulsions. Moreover, in some applications such as biocatalysis, the activity of the biomacromolecules (*e.g.*, proteins, enzymes) is enhanced in an all-water system because their activity is significantly reduced at the oil–water interface.^12,13^ However, stabilisation at the W/W interface remains a challenge due to significantly lower interfacial tension.^14^ Various bioderived materials, such as polysaccharides,^15^ and protein^13^ macromolecules, can be effectively utilised as Pickering particles to stabilise these W/W emulsions.

Although numerous reviews have already been published on the role of inorganic-particle-stabilised Pickering emulsions,^16–18^ their relatively poor biodegradability and biocompatibility significantly limit their practical applications in many fields. With increasing demand for the use of safer and sustainable materials in many applications, recently the use of natural and biodegradable organic solid particles in Pickering emulsion formulations has increased significantly. For example, various polysaccharides and natural proteins have been widely investigated recently under various conditions, such as concentration, temperature, pH and ionic strength. Therefore, this review highlights more recent work (2018 to 2021) using bio-derived materials as stabilisers, focusing on sustainable particles, particularly polysaccharide and protein-based particles used in Pickering emulsions for various applications.

## 2. Polysaccharides in Pickering emulsions

Cellulose, starch, chitosan and chitin are the most common polysaccharide materials. They offer many advantageous features, including their biodegradability, biocompatibility, and low-toxicity and have been utilised in various Pickering emulsion formulations. These particles are already well-established in their applications in food, cosmetics, agrochemicals, pharmaceuticals, regenerated medicines and drug delivery sectors.

### 2.1 Cellulose

**Fig. 3:**
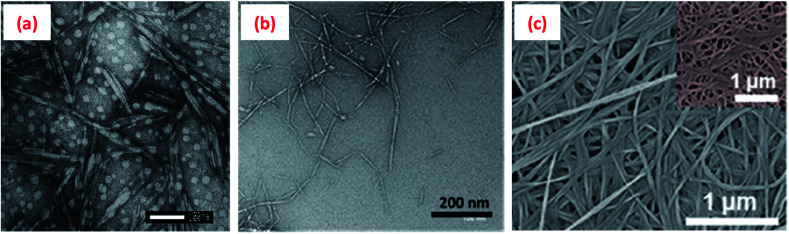
Transmission electron microscopy (TEM) images of (a) cellulose nanocrystals (CNCs isolated from cotton *via* a sulphuric acid hydrolysis process),^29^ (b) cellulose nanofibrils (CNFs obtained from wood pulp utilising a high-pressure homogenisation process)^30^ and (c) scanning electron microscope (SEM) image of bacterial cellulose.^31^ Reprinted from (a) ref. 29, Copyright 2020, under the terms of the CC BY License, published by Wiley-VCH GmbH, (b) ref. 30, Copyright 2020, with permission from Elsevier and (c) ref. 31, Copyright 2020, with permission from American Chemical Society.

Cellulose is generally insoluble in water, but can undergo various chemical and/or mechanical treatments to reduce its size to nanoscale dimension either in the form of cellulose nanocrystals (CNCs) or nanofibrils (CNFs) (Fig. 3a and b). Such treatments generally also impart a surface charge to the cellulose particles, allowing them to be water dispersible. CNCs, mainly rod/needle-shaped particles with a high crystallinity (∼90% crystalline), are usually isolated from various sources (wood pulp, cotton, jute, hemp, ramie, flax, *etc.*) by selectively removing the amorphous regions *via* a controlled acid hydrolysis process. The sizes of CNCs vary between 5-10 nm in diameter and 100–300 nm long, depending on the cellulose sources and hydrolysis process parameters such as type and concentration of acid, hydrolysis time, and temperature.^19–21^

CNCs with comparatively shorter length have better emulsification efficiency as the smaller sized CNCs are favourable for high coverage of oil droplets.^22^ The high coverage is probably obtained *via* side by side stacking of these particles, as well as longitudinal alignment of the self-assembled CNC particles at a relatively higher concentration.^23^ Usually, a form of shear is applied *via* a homogeniser^24^ or ultrasonicator^25^ or microfluidiser^26^ to disperse one phase in another and adsorb rod or fibrillar-shaped cellulose particles at the oil–water interface to form a Pickering emulsion. For example, CNCs have been used to produce low viscosity (liquid-like) O/W Pickering emulsions with precise control over the droplet sizes (around 1 to 3 μm) and high uniformity *via* a high energy microfluidiser.^26^ The emulsion stability can be controlled by the surface charge density of the CNCs since the repulsive forces between particles prevent droplet coalescence. However, an excessive surface charge density (>0.03 e nm^−2^) on CNCs can weaken their adsorption at the oil–water interfaces.^27,28^ On the other hand, CNCs with a surface charge density of below 0.03 e nm^−2^ are found to form stable Pickering emulsions even under an applied external force field (such as centrifugation at 4000*g* for 10 min).^27,28^

**Fig. 4:**
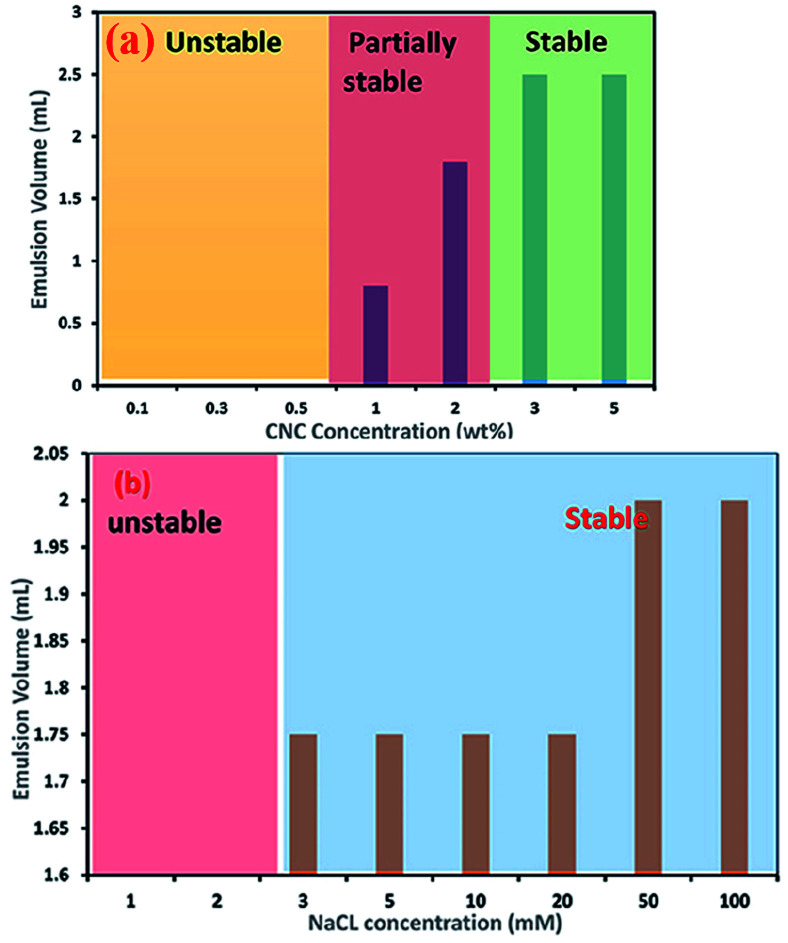
Effect of (a) CNC concentration and (b) addition of NaCl to a 0.1 wt% CNC suspension on the stability of O/W Pickering emulsions.^32^ Reprinted from ref. 32, Copyright 2018, under the terms of the CC BY License, published in the Frontiers in Chemistry.

CNC-stabilised Pickering emulsions have also shown excellent stability against flocculation during changes in various environmental conditions such as temperature, ionic strength, and pH. However, in strongly acidic conditions (pH below 2) and higher ionic strength (for example, 100 mM Na^+^), CNC-stabilised Pickering emulsions show a gel-like behaviour. A considerable amount of protonation (at lower pH condition) and the presence of counter ions (dissociated from the salt) reduces the electrostatic repulsion forces between droplets, allowing formation of a partially flocculated gel network. This phenomenon can also be explained by the change in charge density of the cellulose nanoparticles. Higher concentrations of CNCs in aqueous dispersions with a high surface charge density usually form a stable Pickering emulsion (as can be seen in Fig. 4a, which was assessed *via* measuring the emulsion volume after centrifugation at 4000*g* for 10 min).^32^ At higher concentrations (usually >1 wt%), a significant amount of CNC aggregates adsorb at the oil–water interface, which minimise the interfacial free energy *via* reduction of the interfacial area between the oil–water interfaces to stabilise the emulsion. Adding even a small amount of salt to a Pickering emulsion stabilised by a higher concentration of CNCs can lead to destabilisation of droplets due to aggregation of the surrounding gel networks caused by screening of the electrostatic repulsion forces between the CNCs.^32^ However, Pickering emulsions prepared using a lower concentration of CNCs can be electrostatically stabilised using mono- and divalent salts. For example, stable Pickering emulsions of canola oil in water could be formed at 0.1 wt% CNC with addition of around 3 mM of Na^+^ (see Fig. 4b) or 1 mM of Ca^2+^ ions, suggesting the amount of CNC required to stabilise oil droplets in this Pickering emulsion was reduced by 30% when salt was added.^32^

Regardless of the nanocrystal size, shape and surface charge, the free hydroxyl groups present on the cellulose surface also allow different chemical reactions (such as oxidation or cationisation) to be used to tweak their hydrophilic/hydrophobic balance profile thus imparting favourable wetting properties, vital for stable Pickering emulsion formation. Unmodified cellulose is very hard to disperse in the water phase due to the lack of required surface charge and thus quite challenging to use to form a stable Pickering emulsion. CNCs are covered in anionic sulphate groups resulting from the acid hydrolysis process, rending them hydrophilic and water dispersible. However, the crystalline edge corresponding to the 200(β)/220(α) of the cellulose chain forms a hydrophobic face on crystalline CNC nanoparticles, which facilitates their adsorption at the oil–water interface.^28,33^ Also, CNCs with different crystalline allomorphs exhibit different hydrophilicity. The water contact angles for CNCs-I and CNCs-II are 44.1° and 26.9°, respectively.^34^ Due to their higher hydrophobicity, CNC-I stabilised Pickering emulsions are more stable against centrifugal forces (4000*g* for 2 min) than the CNC-II stabilised emulsions.^34^

Various hydrophobic modifications such as oxidation, esterification or graft polymerisation can also be employed to improve the hydrophobicity of CNCs, thus promoting better adsorption at the oil–water interface for stable Pickering emulsion formation.^35–38^ For example, the water contact angle of octenyl succinic anhydride (OSA) modified CNCs has been reported to be increased compared to unmodified CNCs, to 85.0°, and these particles were effectively utilised to stabilise O/W Pickering emulsions.^35^ Such modifications do not significantly increase the toxicity of the polysaccharides,^39,40^ since they are used in food additives. Amphiphilic CNCs can also be produced by grafting hydrophobic molecules *via* reductive amination for more effective stabilisation of Pickering emulsions.^38,41^

**Fig. 5:**
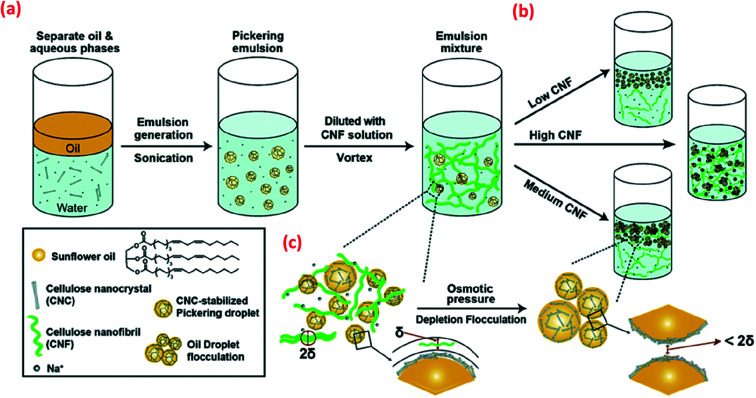
Schematic illustration of (a) the CNC-stabilised Pickering emulsion droplets that are further stabilised by the addition of CNF, (b) with various CNF concentrations, different stabilisation regimes are shown, and (c) Depletion flocculation of oil droplets in the CNC-stabilised Pickering emulsions is shown with non-adsorbed CNF reaching the critical flocculation concentration. The symbol δ in this scheme is used to indicate the size of single CNF or CNF flocs in the aqueous phase.^42^ Reprinted from ref. 42, Copyright 2018, with permission from the Royal Society of Chemistry.

Apart from covalently bound chemical modifications, the stability of CNC-based Pickering emulsions can also be improved by incorporating additional polymers or particles *via* physical adsorption. For example, the addition of non-adsorbed CNFs to CNC-based Pickering emulsions is suggested to adjust the interfacial behaviour *via* a depletion flocculation mechanism (Fig. 5) and thus improve overall emulsion stabilisation.^42^ Depletion flocculation in an emulsion system is associated with an increase of attractive interactions between the droplets, and these attractive interactions can be induced *via* the exclusion of some CNFs from the confined regions surrounding the oil droplets (Fig. 5c). At the CNF critical flocculation concentration, a concentration gradient is created between the bulk suspension (with a high CNF concentration) and the depletion zone (low CNF concentration around the oil droplets), which influences the droplet attraction (mainly originating from osmotic pressure changes between droplets) resulting in droplet flocculation. However, increasing the CNF concentration still further resulted in the formation of a CNF gel, resulting in a lack of droplet creaming, although both flocculated and stable oil droplets were present.

In comparison with CNCs, CNFs are longer fibrillar structures with a substantial aspect ratio (length around a few hundreds of nanometres and a cross-section below ten nanometres)^43^ which are processed utilising various mechanical disintegration approaches, mainly *via* high-pressure homogenisation, ultrasonication and grinding.^44–46^ Usually, CNF production *via* a mechanical process is associated with high energy consumption.^44,47^ Also, most of the fibrils obtained *via* mechanical treatment are in the form of bundles of fibrils rather than the individual fibre. Cellulose nanoparticles having a lower surface charge (for example, *ζ*-potential values between ±30 mV) possess weak mutual electrostatic repulsion forces, which leads to the formation of aggregated networks.^23^ Therefore, a chemical pre-treatment on the source cellulose is generally required before the mechanical defibrillation process to cut the processing time (thus, reduce the energy consumption) and also to impart the necessary charge on the cellulose surface to aid the individualisation of the nanofibrils at a relatively dilute concentration due to the repulsive forces. For example, 2,2,6,6-tetramethylpiperidine-1-oxyl radical (TEMPO)-mediated oxidation of hardwood celluloses is commonly used, prior to processing *via* a high-pressure homogeniser, to produce oxidised cellulose nanofibrils (OCNF).^48,49^ The TEMPO-mediated oxidation process allows the selective conversion of C6-primary hydroxyl groups on the cellulose surface to C6-carboxyl groups, thus increasing negative surface charge.^49^ The *ζ*-potential value of the TEMPO-mediation OCNF (with the degree of substitution ∼25%) is around −60 mV (ref. 50 and 51) and their typical dimensions are around 3–5 nm in diameter and several microns in length.^52,53^

Anionic CNFs are suggested to form an inter-connected fibrillar network at the oil–water interfaces during Pickering emulsion formation.^51,54^ In addition, relatively long fibrous CNFs may adsorb slowly to the droplet surfaces during homogenisation and form relatively thick interfacial layers. Shell thicknesses above 100 nm with a hydration layer have been reported.^51^

Greater adsorption of CNF at the oil–water interface with improved emulsion droplet stability can be governed by increasing the oil–water interfacial tension as investigated using various types of oils.^55^ The amount of CNFs adsorbed on the droplets also increases the interfacial area, calculated using the diameter of the oil droplets.^55^ However, the dispersion stability of CNFs-stabilised droplets depends on the critical concentration of CNFs (in the aqueous phase) rather than those adsorbed on the droplets. Above this concentration, the CNFs form interconnected networks in the water phase resulting in effective stabilisation against creaming, as demonstrated by Bai *et al.*^42^ in Fig. 5.

Since the anionic CNFs possess a highly negative charge on their surface (*ζ*-potential ∼ −60 mV),^50^ emulsion droplets stabilised using these particles also experience electrostatic repulsion with the surrounding droplets, which can be screened by the addition of salt.^51^ For example, the addition of NaCl salt (0.5 M) to an anionic CNF-stabilised Pickering emulsion lowered the surface charge of the emulsion droplets, as the *ζ*-potential values decreased from −46 to −5 mV which resulted in droplet aggregation as confirmed *via* confocal microscopic images.^51^ On the other hand, cationic CNFs (functionalised using glycidyl trimethylammonium chloride with degree of substitution ∼23% and *ζ*-potential ∼ +37 mV) used to stabilise a Pickering emulsion had very little response to salt, as the *ζ*-potential of the droplets decreased only from +24 to +13 mV for emulsions after addition of NaCl (0.5 M).^51^

Bacterial cellulose nanofibres (BCN), with a diameter ranging from 25–100 nm and length from 100 nm to several micrometres (Fig. 3c), are biosynthesised by bacteria in their aqueous culture medium.^56^ Sulphuric acid hydrolysed BCNs possess a higher surface charge (*ζ*-potential −34.8 mV)^57^ than the native fibres and can be well-dispersed in aqueous phase to form a flocculated fibrillar network, which is favourable to stabilise Pickering emulsions.^57,58^ Like CNFs, BCNs are also suggested to adsorbed at the oil–water interface at a higher concentration to form a stable Pickering emulsion.^57^

**Fig. 6:**
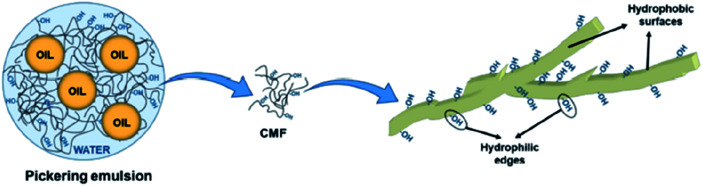
Role of cellulose microfibrils in O/W type emulsion formulation.^59^ Reprinted from ref. 59, Copyright 2019, under the terms of the CC BY License, published by MDPI.

Apart from CNCs and CNFs, micron-sized fibres, such as cellulose microfibres (CMFs), can also be used as emulsion stabilisers by providing a denser packing of fibrils (usually by increasing the concentration of the micron-sized fibrils) which acts as a solid mechanical steric barrier around the droplets to prevent coalescence and creaming (Fig. 6).^59^ The stability of this type of emulsion was however considered more likely to be due to the formation of a gel-like microfibril network around the droplets rather than the adsorption of the cellulose particles at the oil–water interface.

### 2.2 Starch

Starch, the second most abundant polysaccharide after cellulose, is also extensively used to stabilise Pickering emulsions for multiple purposes, mainly in the food industries. Starch consists of amylose (linear glucose units) and amylopectin (branched glucose units) which can form both soluble polymers and insoluble particles in water. The dispersibility of starch in water depends on the ratio of amylose and amylopectin present in the starch, which depends on its source. Common sources include potato, corn, rice, wheat, maise, and cassava starch.^60^ Therefore, the emulsification ability of starch depends on the starch source and type. Starch particles can adsorb on oil droplets and also form an inter-droplet network leading to gel-like behaviour in the Pickering emulsions. However, starch in its native granular form is not hydrophobic and hence not suitable to adsorb at the oil–water interface during the emulsification process. The hydrophobicity of starch can be increased by chemical or physical modification. Starch molecules can be chemically modified *via* esterification with octenyl succinic anhydride (OSA) to improve their hydrophobicity and thus increase the affinity of the starch particles to oil.^61–64^ Although the particle size distribution of the native starch is not significantly influenced by the OSA-esterification;^65^ the hydrophobic modification in turn, resulted in smaller droplet sizes in the emulsion, slower creaming and better stabilisation of the Pickering emulsion than emulsions prepared with unmodified starch.^62,66^ In addition, OSA-modifications are not considered as toxic since they are used in food additives.^39,40^

**Fig. 7:**
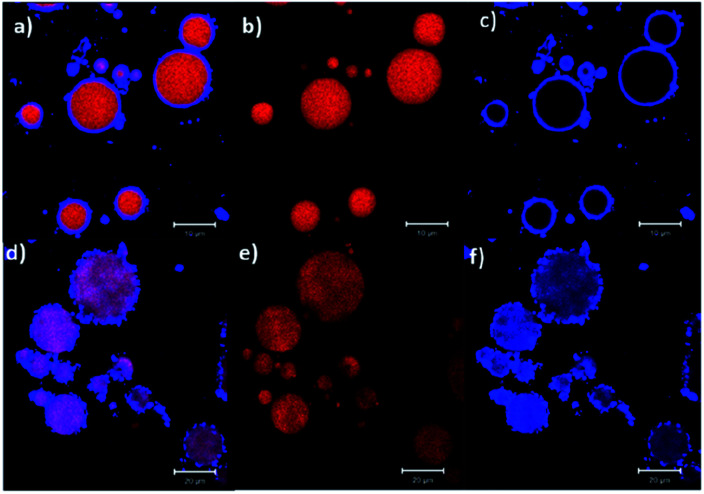
Confocal micrographs of Pickering emulsions prepared with (a–c) 20% wt of native starch, and (d–f) 20% wt of modified starch (esterified with lauroyl chloride).^66^ Reprinted from ref. 66, Copyright 2018, with permission from Elsevier.

Confocal microscopy images of starch-stabilised Pickering emulsions are shown in Fig. 7 (blue regions correspond to starch and red areas are those of the oil phase), where the droplets of emulsion prepared with unmodified native amaranth starch show a lower coverage of the oil phase compared to those prepared with modified starch (esterified with lauroyl chloride).^66^ Such hydrophobic modification of starch can also lead to the formation of aggregated particles in the aqueous phase and which adsorb at the oil–water interface as aggregates rather than individual particles providing local larger thicknesses of starch on the oil droplets at the adsorption sites (Fig. 7).^66^

Decreasing starch particle size also tends to decrease Pickering emulsions' droplet size while increasing storage stability.^67^ The decreased particle size provides a better cohesive barrier at the oil–water interface due to more efficient packing. Different processes, such as acid hydrolysis,^68^ non-solvent precipitation,^69^ ultrasonication, and media-milling,^70^ are commonly used to reduce the starch particle size. However, in terms of sustainability it is preferred to avoid using corrosive acids, toxic chemicals and ultrasonication processes (which utilise high energy and are limited to small volume production). Hence, less toxic chemical treatments along with milling or high-pressure homogenisation processes can be employed for large scale production of reduced sized starch particles in a more sustainable way.

### 2.3 Chitosan

Chitosan is a linear polysaccharide obtained by the deacetylation of chitin, naturally occurring in the exoskeletons of crustaceans, insect cuticles and cell walls of some fungi. Chitosan possesses antibacterial, antifungal, mucoadhesive and gelling properties, making it a promising material for many industrial applications, especially candidates in tissue engineering and drug delivery, although concerns about allergic reactions to marine proteins can limit its use in such products. Chitosan is usually soluble in a dilute acidic medium due to the protonation of the free amino groups on their surface, which leads a weak surface activity resulting in the poor emulsifying capacity. Usually, by increasing the pH (pH > p*K*_a_) close to neutral, deprotonation of NH_3_^+^ group to NH_2_ results in a decrease in the net charge of the chitosan molecules, which increases their intermolecular attraction and intensifies the hydrophobicity to form self-aggregated chitosan particles (with mean diameter ∼287 nm and *ζ*-potential ∼ +24 mV).^71^ Chitosan has been investigated to stabilise Pickering emulsions by increasing the continuous phase viscosity or by forming a layer (*via* self-aggregation)^71^ at the surface of the dispersed oil droplets. In addition, the hydrophobicity of the chitosan particles can also be tailored with the addition of various oppositely charged surface-active agents such as polyanions,^72^ proteins^73^ or other polysaccharides^74–76^ forming complex coacervates where the hydrophilicity can be tuned to improve emulsifying capacity.

**Fig. 8:**
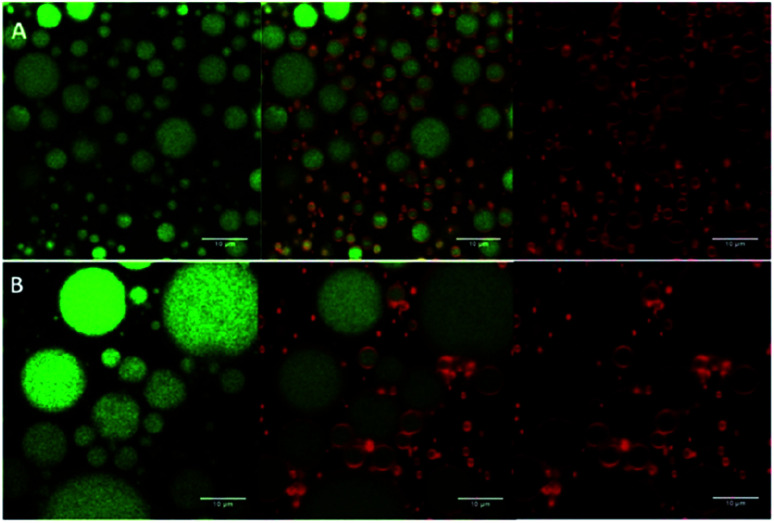
Confocal laser scanning microscopy images of Pickering emulsion formulations prepared with a fixed concentration of chitosan/gum arabic (1 : 1) nanoparticle dispersion (1.5% w/v) and an oil volume fraction of (A) *φ* = 0.5; and (B) *φ* = 0.7. The emulsion oil phase appears in green (on the left), whereas the adsorbed nanoparticles appear in red (on the right). The images in the middle are an overlay of these two images (right and left).^76^ Reprinted from ref. 76, Copyright 2019, with permission from Elsevier.

Pickering emulsions prepared from chitosan/gum arabic (1 : 1) dispersions (1.5% w/v, with average diameter ∼108 nm and *ζ*-potential ∼ +56 mV), and high oil volume fractions (*φ* = 0.5, 0.7), have shown good storage stability *via* the effective adsorption of the complex particles (formed *via* electrostatic attraction between the positively charged chitosan and negatively charged gum arabic) at the oil–water interface, forming a barrier against droplet coalescence (see Fig. 8).^76^ Moreover, by increasing the oil volume fraction and the concentrations of the particles, gravitational phase separation during long-term storage was inhibited.

## 3. Proteins in Pickering emulsions

Proteins are one of the most commonly used classes of stabilisers for Pickering Emulsions, so the literature around the use of protein particles for Pickering emulsion is extensive and includes applications such as drug delivery,^77^ biocatalytic activity^12,13,78^ and encapsulation of nutritional agents like Vitamin D_3_.^79^ Current research is focused on a wide variety of protein-based Pickering stabilisers with differing tuneable characteristics. The stabilisation of the emulsion works through steric repulsion of the particles as well as strong electrostatic repulsions between the charged surface-active particles. The inherent bioderived nature of proteins makes them an attractive edible choice for the food industry which has been discussed in depth in recent reviews.^80–82^ With a rise in vegetarianism, veganism and for religious reasons, there has been growing demand from consumers for emulsion-based protein products that are not sourced from animals. This has driven a greater focus on investigating the emulsification properties of various food-grade plant proteins such as pea protein,^83^*Persea americana* Mill^84^ (a by-product of avocado oil processing) and peanut protein^85,86^ as replacements for animal-based proteins. These plant proteins can be more sustainable, have excellent emulsion capabilities and are usually less expensive than more commonly used dairy proteins, however are usually less soluble in aqueous solutions and are less digestible.^83^

Protein-based emulsion stabilising particles can be broken down into two key classes – ‘nanoparticles’ and ‘gel network particles’, although there are other shapes including nanofibrils and nanocages, which were discussed in depth in a recent review,^81^ so these will not be included here. Protein nanoparticles have various simple preparation methods including emulsification, complex coacervation and electrospray. Due to their ease of adsorption at the oil–water interface and cost effectiveness, nanoparticles have dominated most previous protein-based Pickering emulsion studies. Protein nanogel particles however are now of increasing interest to form responsive Pickering emulsions, so both of these classes of protein-based Pickering stabilisers are discussed below.

Curcumin, a lipophilic polyphenol derived from the turmeric plant, has been suggested to have many health benefits such as being an anticarcinogenic, anti-inflammatory and antioxidant. However, due to its high hydrophobicity, chemical instability, and poor bioavailability, it has not been fully utilised in modern medicine and research has escalated towards finding the right delivery vector to encapsulate the nutraceutical for ingestion. Curcumin encapsulation is used here as an example to demonstrate the potential of a variety of protein-based systems for Pickering emulsion stabilisation and delivery of such actives. Pickering emulsions have shown excellent potential to improve the oral bioavailability of lipophilic species with the physical barrier at the interface of the droplets able to protect the active species from physiologically harsh conditions. For example, ovotransferrin fibrils, a glycoprotein derived from eggs, have been investigated as an efficient Pickering stabiliser that can also protect encapsulated curcumin from UV degradation.^87^ Increasing the salt concentration present in solution to 1000 mM and adjusting to pH 6 gave the emulsion the best stability and UV protection as it was hypothesised that with a reduced electrostatic repulsion, the fibrils could form a denser, thicker shell around the encapsulated curcumin. While these optimal salt and pH conditions are obviously unrealistic for physiological uses, the bio-accessibility of curcumin increased up to 129% using a TIM-1 digestion model under more realistic physiological conditions showing the potential that ovotransferrin fibres hold for future uses as a means of encapsulating curcumin.

Pickering emulsions have also been explored to influence a higher level of organisation within a system, using the stability of the particle stabilised emulsion to embed it in a hydrogel network. Double cross-linked zein–sodium alginate emulsion gels, using a Pickering emulsion template, with a dense network microstructure and high viscoelasticity were able to encapsulate two nutraceuticals – curcumin and resveratrol.^88^ The double cross-linked system contained transglutaminase-linked zein nanoparticles adsorbed to oil droplets, to form a Pickering emulsion, which were then embedded in a calcium alginate cross-linked network. This gave an emulsion gel with superior nutraceutical bio-accessibility and light stability in comparison to a single cross-linked system. The adsorption of the anionic biopolymer, sodium alginate, provided additional protection against coalescence of oil droplets by increasing the electrostatic and steric repulsion between them and an increased concentration of transglutaminase led to stronger gel formation through further cross-linking. Modifying the protein surface using biopolymers such as carboxylmethyl dextran^89^ has been shown to also increase the bio-accessibility of curcumin in protein-stabilised Pickering emulsions by reducing the hydrophobicity of the protein surface. Other hybrid particle complexes have also been investigated in relation to curcumin encapsulation including gliadin (protein)–lecithin (phospholipid),^90^ whey protein isolate (protein)–lactose (polysaccharide)–epigallocatechin gallate (polyphenol)^91^ and phosphatidylcholine (phospholipid)–kaolinite (clay).^92^ This wide variety of complexes demonstrates the versatility and tunability of protein based particles for Pickering emulsion stabilisation and the potential for using the Pickering stabilised emulsion droplets as building blocks in more complex delivery systems.

High Internal Phase Emulsions (HIPEs) have also received increased interest in recent years. They are defined as having an internal phase volume of at least 74% and in the past have commonly required a high concentration (5–50 wt%) of surfactants to prevent flocculation of the droplets, which can be considered a potential environmental problem. Research has therefore turned to High Internal Phase Pickering Emulsions (HIPPEs) as an alternative. Above this defined volume, the oil droplets tend to deform from spherical to polyhedral in shape to increase the packing and the emulsion becomes highly viscous. Inorganic particles such as silica^93^ and titania^94^ have been explored to stabilise HIPPEs but the potential release of non-degradable particles has led to a shift in focus to food grade biopolymer-based particles such as peanut protein isolate,^86^ perilla protein isolate^95^ and casein nanogels^96^ because of their biocompatible nature. A large benefit of the high oil volume capacity of HIPPE is that the emulsion has a greater loading capacity for hydrophobic compounds during encapsulation, however, there is the risk of phase inversion within the emulsion at such high oil volumes.

HIPPEs have the potential to reduce the need for partially hydrogenated oils (PHOs) in food products by forming solid-like fats from highly concentrated liquid oils. However, common protein particles *e.g.* zein and whey protein isolate (WPI) tend to rearrange at the biphasic interface when used as the sole stabiliser leading to lower stability of the emulsion. This has led to a search for novel proteins to use as a sole stabiliser for HIPPEs with Zhao *et al.* pioneering the use of perilla protein isolate, a by-product residue from the processing of perilla oil.^95^ Initial studies found the defatting methods to isolate the protein residues significantly impacted its functional properties such as foamability and solubility. Cold-pressed residues displayed the most promising properties, albeit at high pH, such as small droplet sizes (27.55 μm), high foaming ability (90.67% at pH 11) and good emulsification ability (3390.1 m^2^ g^−1^ at pH 11), for future use as Pickering stabilisers in comparison to hot pressing and solvent extraction methods. The improved performance was due to the protein particles forming at a smaller size (318 nm), with high hydrophobicity, the largest electrostatic repulsion, and a higher solubility than alternative defatting methods. However, the oil fraction limit for the emulsion was capped at *φ* = 0.5 (ref. 97) so this group went on to combine the particle preparation method of pH cycling (which can give a heterogeneous distribution of particle sizes) with further homogenisation through high speed shearing to give perilla protein nanoparticles (PNPs) that could form HIPPEs with a gel-like network and an oil fraction up to *φ* = 0.75 with as small PNP concentration as 1%.^95^ Despite this improvement, future work must continue to increase the oil fraction dispersed by the particles to be seen as a viable option as a HIPPE which is competitive with current surfactant-based technologies. Other groups are focused on casein nanogels^77^ and egg proteins^98^ to achieve viable HIPPE systems.

Microgels and nanogel Pickering particles, of micrometre and nanometre size respectively, have also recently been brought to the forefront of research. They are soft colloidal particles that hold the ability to swell upon solvent addition and are prepared commonly through top-down methods. A cross-linked hydrogel is formed from a concentrated protein source and then homogenised and broken down into smaller gel particles under shear. Three-dimensional gel networks are formed when there is a high concentration of non-adsorbed particles with strong electrostatic forces from the protein functional groups keeping the network rigid and have been shown to improve the stability of emulsions as the oil droplets cannot aggregate due to the network rigidity, giving them semi-solid characteristics. Nanogel particles have been used much less commonly as Pickering stabilisers in comparison to microgels, with few examples in literature in recent years.^77,99,100^

A protein by-product of the cheese industry, whey protein isolate (WPI), has previously been used as a stabiliser both alone^101^ and as a hybrid with other biopolymers.^91,102^ It is limited in its use due to its tendency to denature during the pasteurisation stage. Whey protein microgel (WPM) particles can be formed when WPI is heated to high temperatures^103^ or subjected to hydrostatic pressures^104^ at a pH close to the isoelectric point (pH 5.0–6.0).

**Fig. 9:**
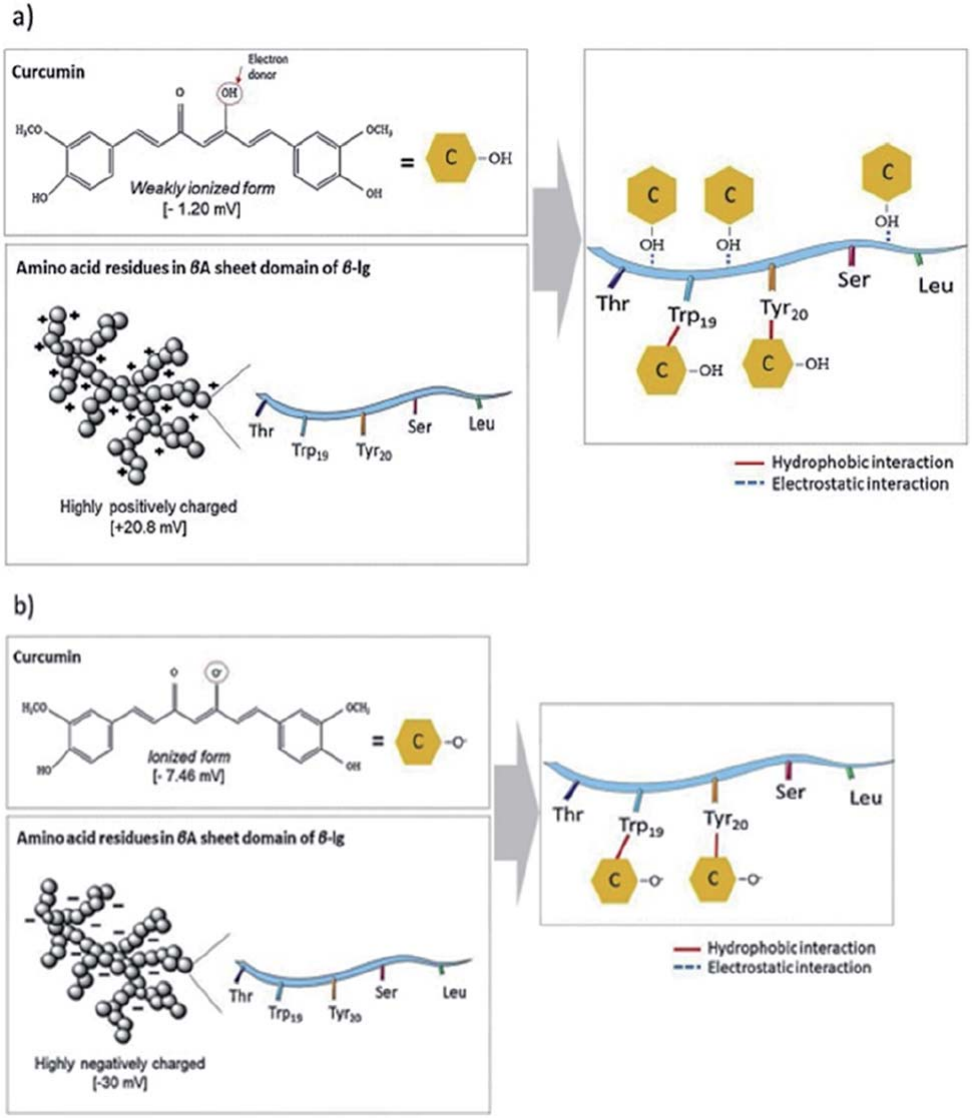
Schematic representation of the non-covalent binding interactions of whey protein nanogel with curcumin at (a) pH 3.0, and (b) pH 7, respectively. The grey spheres represent WPN and yellow hexagons represent curcumin.^99^ Reprinted from ref. 99, Copyright 2019, with permission from Elsevier.

**Fig. 10:**
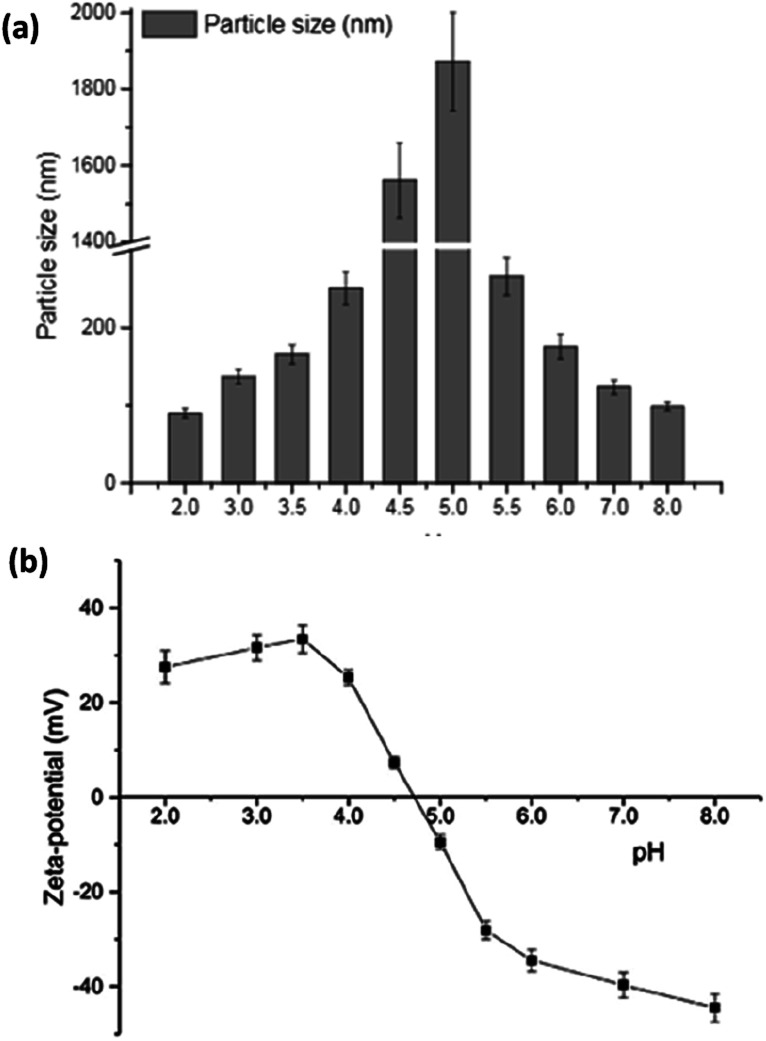
The mean particle size (a) and *ζ*-potential (b) of whey protein isolate (WPI) gel particles, produced using high hydrostatic pressures, as a function of pH.^104^ Reprinted from ref. 104, Copyright 2020, with permission from Elsevier.

Smaller whey protein nanogel (WPN) particles, with 83.05 ± 1.74 nm particle diameters, formed by passing a heat-set hydrogel through a high pressure homogeniser twice, have been used as a Pickering stabiliser for curcumin encapsulation where the small, nanometric sized gaps between the particles (∼30 nm) at the droplet interface prevented curcumin diffusion out of the oil droplet.^99^ The 500 μg ml^−1^ of curcumin within the droplets was shown to bind through hydrogen bonding and electrostatic interactions to the nanogel particles and remained stable under physiologically relevant conditions, showing promise for use as a future delivery vector.^99^ Varying the pH of the system greatly altered the binding affinities and binding mechanisms of the curcumin to the WPN particles as shown in Fig. 10. At lower pH values, the amino acid residues in WPN are positively charged leading to strong electrostatic attractions with the weakly negative charge of the curcumin (Fig. 9a). However, at higher pH values, the main form of bonding interaction is from hydrophobic interactions as both the WPN and curcumin possess negative charges (Fig. 9b). The difference in gel particle size between nano- and micro-examples formed under hydrostatic pressures has been attributed to small differences in preparation routes with a highly localised pressure, less turbulent flow and a lower protein concentration in the heat set hydrogel seeming to lead to smaller nano-sized gel particles.

HIPPEs stabilised solely by protein particles can also be improved upon by using the protein microgel particle counterpart as the Pickering stabiliser.^103^ In comparison to native non-gelled WPI particles and a standard surfactant (Tween 20), HIPPEs formed using WPM particles had a better thermal stability, greater shelf life and were overall more viscous.^103^

During particle preparation using high hydrostatic pressure, the diameters of the WPM particles varied greatly depending on the pH of the aqueous conditions.^104^ As the pH approached the isoelectric point (pI), the *ζ*-potential of the gel particles reached a point of zero charge. This led to less electrostatic repulsion occurring (Fig. 10b) and micrometric sized particles forming (Fig. 10a) around the pI as opposed to the nanometric sized particles both above and below this pH. The combination of lower electrostatic repulsion and larger particles produced a tighter internal structure, and more rigid packing at the interfaces, ideal stabilisers for formation of Pickering emulsion gels that had a lower release profile for the nutraceutical curcumin than liquid Pickering emulsions. This same particle size trend occurred for both peanut protein^86^ and pea protein^83^ microgel emulsions as aggregates formed at the isoelectric point. Crosslinking WPM particles with organic acids, such as citric and tannic acid, has been shown to produce smaller, less polydisperse particles that also appeared to offer a better emulsion stability compared to conventional WPM stabilised emulsions.^105^

Protein nanoparticles and micro or nanogels are therefore promising bioderived materials for future studies with a large range of possible protein sources, especially considering the potential for valorisation of waste generated in other food processing steps.

## 4. Lipid nanoparticles

The term lipid is used to encompass a large and diverse range of molecules that retain the key feature of organic compounds that are insoluble in water. Edible lipid particles have become increasingly studied as Pickering particles due to their biocompatibility and ease of production. This section will focus on recent trends involving solid lipid nanoparticles and oleofoams.

### 4.1 Solid lipid nanoparticles

Polymer nanoparticles have commonly been used as a drug delivery encapsulation option, however the cost of production and regulatory restrictions on safety has led to research into alternatives such as Solid Lipid Nanoparticles (SLNs) which offer a reduced toxicity and better biocompatibility. It has been proposed that SLN-stabilized Pickering emulsions could act as temperature sensitive nano-carriers, with ketoprofen used as a model hydrophobic drug solubilised in medium chain triglycerides (MCT).^106^ The nano emulsion was stabilised using 40 nm sized SLNs and at physiological temperatures (37 °C), there was partial melting of the SLNs which led to a slow drug release as the barrier between the oil and the aqueous phase weakened. A recent proof-of-principle study also explored the potential of co-encapsulation and co-delivery of hydrophobic and hydrophilic active ingredients within the same emulsion.^107^ First, a model hydrophobic active ingredient, Sudan III, was encapsulated within the SLN itself then depending on the type of emulsifier used to stabilise the SLNs, dimethylphthalate (DMP) and NaCl were encapsulated within a O/W and W/O emulsion respectively.^107^ The potential for multi modal drug delivery of both hydrophobic and hydrophilic actives is a highly desirable concept, diminishing the need for multiple doses as well as offering potential synergistic benefits.

The inherent hydrophobicity of the lipid particles leads to the need to incorporate small amounts of surfactants or other emulsifiers in the formulation stage to tune the hydrophobic nature and minimise coalescence of droplets. This makes it an option that cannot feasibly be surfactant-free but there is a reduced toxicity risk in comparison to a system stabilised exclusively by surfactants due to the lower concentrations. It has been shown that there is a strong correlation between the type and concentration of emulsifiers used during formulation of the SLNs and the interfacial properties of the particles formed.^108,109^ Recent studies showed the De Brouckere (D_4,3_) mean diameters of SLNs reduced in size significantly with the increasing concentration of different PEGylated emulsifiers used in the preparation. Increase of the surface load of the emulsifiers (*Γ*_s_) led to the decrease in contact angle between the SLNs and the oil–water interface, closer to the optimal 90° angle, with the SLN prepared using a Brij S20 emulsifier decreasing from ∼155° to 135° as the surface load increased from 10 to 20 mg m^−2^.^108^

A common method of preparation of SLNs involves heating the lipid phase above its melting point, then addition of the aqueous phase containing small amounts of the emulsifier at the same temperature.^110^ The phases are then emulsified, and the nanoparticles formed, commonly by high shear homogenisation or ultrasonic treatment, which can lead to a more stable emulsion containing smaller droplets with an average diameter typically under 300 nm and a narrow particle size distribution.

**Fig. 11:**
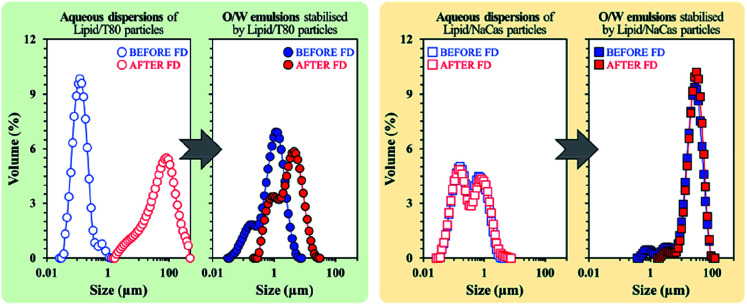
Particle size distributions of SLN particles stabilised by Tween 80 (left) and sodium caseinate (right), before (blue) and after (red) lyophilisation.^111^ Reprinted from ref. 111, Copyright 2020, with permission from Elsevier.

Freeze drying (lyophilisation) is a common method in the food industry for preserving perishables through the removal of water before rehydrating at a later date. Zafeiri *et al.* investigated the effect of dehydration/rehydration on SLN-stabilised Pickering emulsions to determine how particle morphology and size changed with varying factors such as lipid type, surface active species type and concentrations of both.^111^ Results showed that in the presence of a low molecular weight surface active species *e.g.* Tween 80, the lipid nanoparticles initially formed were uniform and submicron, in comparison to when a high molecular weight surface active species, sodium caseinate was present, giving larger but still uniform particles. Lipid particles formulated using sodium caseinate however, emerged as the superior choice, retaining the same particle size, microstructure and Pickering functionality with similar emulsion droplet sizes being formed before and after the freeze-drying and rehydration process (Fig. 11). The use of Tween 80 led to significantly increased particle sizes after lyophilisation which in turn led to larger emulsion droplets being stabilised (Fig. 11). It is promising that sodium caseinate, a naturally occurring milk protein, is the more effective stabiliser for this system as it is inherently more sustainable due to its bioderived nature in comparison to the surfactant Tween 80. Future research should look towards seeking out more bioderived SLN stabilisers like sodium caseinate as we move towards a more sustainable future.

### 4.2. Oleofoams

**Fig. 12:**
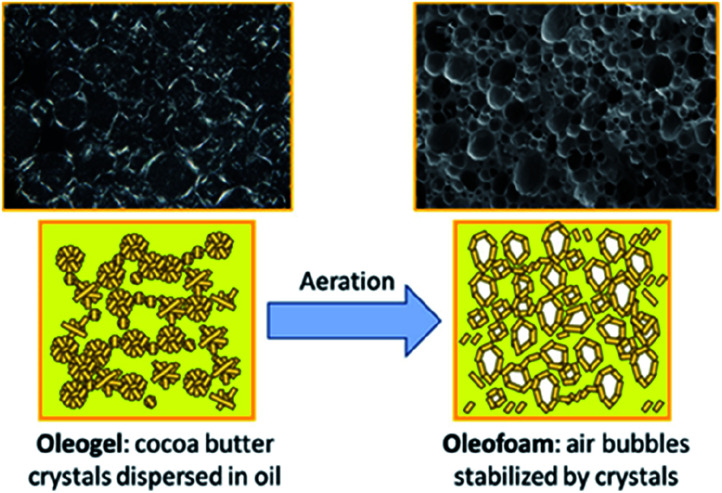
Diagram showing the aeration of a cocoa butter oleogel to form crystal-stabilised air bubbles within the oil, known as oleofoams.^112^ Reprinted from ref. 112, Copyright 2021, with permission from American Chemical Society.

Oleofoams, best described as edible air-in-oil systems,^112^ can be made by the vigorous whipping of oleogels (Fig. 12), which in turn are formed using gelators to create dispersions of lipid, fatty acid or wax crystals in a continuous oil phase. The fat crystals adsorb at the air–oleogel interface *via* a Pickering mechanism and prevent the coalescence of air bubbles.^113^ There has been some debate as to whether oleofoams can be classed as true Pickering emulsions as it is hypothesised that the trapped air bubbles may not only be stabilised by solid crystal particles but also by the slight bridging of crystals to form a strong network stabilisation.^114,115^ We discuss them in this review, however, as it is thought that the Pickering mechanism is critical to the stabilisation of the foam.

Oleofoams are much less common than their aqueous-based foam counterparts, with the low surface energy at the air–oil interface making them more difficult to prepare.^116^ However, they have received increased interest in recent years, particularly in the food industry as they can provide a reduced calorific content whilst still giving an aerated, smooth texture with similar rheological properties to their full-fat counterparts.^112^ Also, in contrast to aqueous foams, the lack of water present leads to less microbial spoilage with less requirement for preservatives.^116^ Research is restricted by the polymorphism and concentrations of fat crystals chosen to investigate since below a certain concentration, the crystals provide insufficient coverage to stabilise the air within the oil whereas above the optimal range, the oleogel becomes too difficult to aerate.

A recent study focused on how the shape, size, concentration and polymorphism of the fat crystals in the initial oleogel affected the microstructure, stability and rheological properties of the resulting oleofoam.^112^ The authors used cocoa butter (CB), high in saturated fats, as their high melting point fat, added to high-oleic sunflower oil at elevated temperatures before cooling. They found the oleofoam to be stabilised by CB nanoplatelets (up to 500 nm in size) in the β(V) polymorph state with an air-volume fraction of up to *φ* = 0.60–0.66. The diameters of the spherical fat crystal aggregates produced in the oleogel increased slightly upon longer cooling times, with the fastest cooled samples, approximately −0.5 °C per minute, being around 40 μm in size. The medium and slow cooled samples, at ∼−0.2 °C and −0.08 °C per minute, had larger aggregates of 50–60 μm and 100–150 μm, respectively. However, it was determined that the cooling rate of the CB crystals was not a defining factor in the overall production of the oleofoams as, during the aeration step, the temperature was raised 5–10 °C so some of the smaller CB crystals were dissolved, changing the size distribution and morphology range of the crystals but giving overall similar properties in the oleofoams from all of the precursor gels.

The overriding factor in the size and shape of the air bubbles was the CB content with 30% CB w/w having a less efficient mean foam overrun and some unaerated oleogel crystals still present in the resulting oleofoam. The samples with lower concentrations of 15% and 22% w/w CB gave superior results with higher foam overrun. This reliance on crystal concentration was also shown by Du *et al.*^115^ as in that work, the increase of monoglyceride concentration led to tightly formed bubbles and stronger interfacial elasticity. This was thought to be due to a thicker crystal layer at the interface and an increased gelling within the continuous oil phase.^115^

Hybrid oleofoams have also been formulated using medium-long chain diacylglycerol (MLCD) and β-sitosterol (St) in varying ratios as the gelator components, interacting through hydrogen bonding.^117^ It was found that MLCD acted as the main Pickering stabiliser whilst St prevented the MLCD crystals from aggregating and synergistically improved the stabilisation and rigidity. The addition of diacylglycerol particles to fully hydrogenated palm oil (FHPO) was also found to form highly stable oil foams with high viscoelasticity through heterogeneous nucleation which gave a strong network system with less chance of oil drainage or air bubble coalescence.^114^

## 5. Hybrid particles in Pickering Emulsions

### 5.1 Protein–phospholipid hybrids

Single component nanoparticles can have restricted particle properties and proteins, in particular, are sensitive to pH, ionic strength and high temperatures, especially at the isoelectric point, which can lead to emulsion instability. However, proteins are arguably the best option for food grade nanoparticles due to their biocompatibility and vast range of surface active functional groups, so recent research has focused on reducing the sensitivity of protein nanoparticles whilst retaining their edibility by combining them with phospholipids to form composites.

**Fig. 13:**
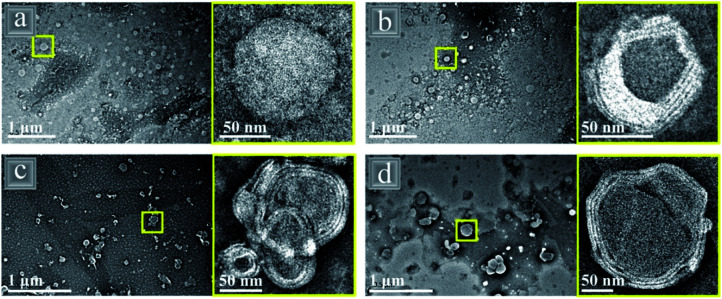
TEM images of gliadin (G)–lecithin (L) core–shell nanoparticles with varying ratios of G : L (a) 10 : 0, (b) 7 : 3, (c) 5 : 5 and (d) 3 : 7.^118^ Reprinted from ref. 118, Copyright 2019, with permission from American Chemical Society.

Several recent studies have combined gliadin (G) protein with the phospholipid lecithin (L) using an anti- solvent co-assembly to form core–shell like nanoparticles with a protein rich core and phospholipid rich multi-layer shell (Fig. 13).^90,118,119^ The smallest nanoparticle diameter of 77.8 nm was formed with the smallest amount (7 : 3 G : L) of phospholipid present, most likely due to small amounts of phospholipid having the ability to lower the surface tension and inhibit aggregation whereas larger amounts would cause bilayers of the phospholipids to form, creating larger diameters. It was found that the higher the phospholipid content of the nanoparticle, the more stable to changes in pH, salt addition and heat the overall emulsion was and it also had an increased foamability.^118^ SDS-PAGE and FTIR confirmed that the composites were held together with hydrogen bonding, with no covalent bonds present and that the order of addition during the antisolvent co-assembly step could affect the interactions and structure of the nanoparticles.^90^ As well as their excellent potential as a foaming agent, the hybrid nanoparticles were used to encapsulate the hydrophobic nutraceuticals curcumin^90^ and quercetin^119^ and reduce their degradation within the body, in comparison to pure gliadin nanoparticle emulsions.

### 5.2 Protein–polysaccharide hybrids

Protein surface modification with polysaccharides can also improve their wetting properties,^120^ resistance to ionic strength, pH and temperature changes^121^ and stability against creaming and flocculation.^120^ By understanding, controlling and modulating the interactions that occur between proteins and polysaccharides, the resulting complexes can give superior functional and emulsifying properties. As proteins can change conformation and zeta potential due to pH, the strength of interaction between the positively charged protein, below the pI (isoelectric point), and the (usually) negatively charged polysaccharide can vary significantly, influencing emulsion properties. A commonly investigated Pickering particle, zein, is a prolamin based protein, rich in hydrophobic amino acid residues, that is a by-product from the cereal processing industry. It can be used without modification as Pickering nanoparticles but due to its highly hydrophobic nature and lack of wettability, especially at pH's close to the isoelectric point, it tends to aggregate easily. Their high hydrophobicity is excellent at encapsulating hydrophobic biomolecules, however, so a common solution to improve the emulsion stabilities is to cross-link the protein with natural biopolymers such as gum arabic,^122^ chitosan^79^ and corn fibre gum.^120^ These biopolymers have been shown to reduce the contact angle and hydrophobicity of the zein particles, improving the emulsifying properties and stability of the Pickering emulsion.

Along with the above mentioned non-covalent interactions to form particle complexes, proteins can also form covalent conjugates through the Maillard reaction, or *via* chemical or enzymatic cross-linking with polysaccharides. A recent perspective paper highlighted how the design of protein–polysaccharide delivery vehicles for bioactive ingredients can be tailored depending on the bonding mechanism of the complex to fit different delivery needs.^123^ It also discussed the challenges that must be overcome in this area including low loading capabilities and the need for targeted and controlled release.^123^ As there is such a wide variety of protein–polysaccharide combinations available, investigation into the effects of different types is ongoing and a selection of recent studies have been summarised in Table 1 to give a flavour of this area of work.

**Table 1:** A summary of protein–polysaccharide combinations used as Pickering emulsion stabilisers

Type of protein–polysaccharide complex	What was encapsulated?	Overview	Advantages	Reference
Hordein–chitosan	Capric triacylglyceride	Complex particles formed *via* hydrogen bonds and hydrophobic interactions, optimal ratio of 2 : 1 hordein : chitosan, improved partial wettability of hordein particles. Pickering emulsions with up to 60% oil fraction were achieved with gel-like networks present at 50% and 60%	• Three-phase contact angle 89.3 ± 1° suggesting good partial wettability	Li *et al.*^124^
			• Chitosan has been used frequently in previous work for its biocompatibility and biodegradability	
Lactoferrin nanogel (LFN)–inulin nanoparticles (INP)	Sunflower oil	Primary Pickering emulsion with lactoferrin nanogel particles. An additional secondary inulin interfacial layer acted as a protective steric barrier, delaying gastric digestion by pepsin. Did not fully inhibit pepsinolysis due to diffusion of pepsin through gaps between nanoparticles	• Additional steric protection for LFN nanogel particles that are sensitive to pepsin	Sarkar *et al.*^125^
			• Inulin is already commonly used in food and pharmaceutical industry	
Ovotransferrin (OVT)–gum arabic (GA)	Medium chain triglycerides	OVT–GA nanoparticle stabilisation achieved through hydrogen bonding, electrostatic attractions, and hydrophobic interactions. Optimal, uniform nanoparticles with contact angles of 82.6° were formed at OVT : GA ratio of 3 : 1 and at pH 3.2 they could adsorb at oil–water interfaces	• The glycoprotein OVT has been shown to have antimicrobial and antioxidant properties	Wei *et al.*^87^
			• OVT is naturally abundant and can be easily dispersed in water without the need for organic solvents	
Pea protein isolate (PPI)–high methoxyl pectin (HMP)	β-Carotene in corn oil	Colloidal particles formed through electrostatic attraction to encapsulate β-carotene within a stable HIPPE with oil fractions up to 80% w/w	• PPI-HMP displayed better stability against pH variation in comparison to PPI-alone emulsions	Yi *et al.*^126^
Soy protein isolate (SPI) with	• Soy protein is an easily sourced and cheap plant based complete protein			
	• SPI has good emulsifying, foaming and gelling properties but is susceptible to pH, ionic strength and temperature change so modification necessary			
	• SPI is low cost and has an ideal amino acid pattern			
– Chitosan	Corn oil	Food grade nanoparticles synthesised to stabilise gel-like Pickering emulsions. Increasing particle concentration led to smaller droplet sizes and increased gel strength	• Stable over broad range of ionic strengths (0–1000 mM) and temperatures (4–60 °C) with little change to droplet size after storage for 20 days	Yang *et al.*^127^
– Pleurotus eryngii polysaccharide (PEP)	Corn oil	Conjugate nanoparticles prepared *via* a Maillard reaction under controlled wet-heating conditions which were then used to encapsulate β-carotene. Conjugates formed through covalent bonding and improved water solubility and emulsion properties between pH 4–11 which improved the bioavailability of β-carotene	• Wet heating method has faster reaction rates and increased glycosylation yields over a dry heating method	Hu *et al.*^128^
			• PEP has multiple health benefits *e.g.* anti oxidant and anti tumour	
– Tempo-oxidised bacterial cellulose (TOBC)	Canola oil/Dodecane	TEMPO oxidation made the cellulose nanofibrils negatively charged to increase electrostatic interactions with SPI. Adsorption of SPI to TOBC increased surface hydrophobicity of the cellulose nanofibrils, so improved rheological properties, interfacial tension, and long-term stability	• Easier and “greener” than alternative surface modification options	Zhang *et al.* (2019)^129^
		The effects of pH, oil fraction and solid content on the stability and emulsion structures formed using SPI/TOBC complexes were investigated. Smallest droplet sizes were achieved at pH 7, with highest TOBC content, however electrostatic complexation between SPI and TOBC was more favourable at pH 3 and gave higher creaming stability. With increasing oil content (up to 74%), the droplet shape of the emulsion began to shift from spherical to polygonal	• Improved oxidative stability and anti-digestibility in comparison to SPI nanoparticles alone	Zhang *et al.* (2020)^130^
		At other pH values, emulsions containing only SPI nanoparticles had the poorest interfacial adsorption around the isoelectric point, pI = 4.5. TOBC significantly improved emulsification and stability by inhibiting interfacial penetration		Zhang *et al.* (2021)^121^
Various proteins–gum arabic (GA)	Soybean oil	Compared the emulsifying and interfacial properties of soy proteins (SP), heat treated soy proteins and gelatin, all complexed with GA. When formed at a 1 : 1 ratio of protein: polysaccharide, SP based (especially the heated treated) systems showed superior emulsification behaviour over gelatin due to difference in protein conformation. The systems were highly pH dependent with turbidity being utilised to determine optimal pH values for each system	• The replacement of gelatin with SP would reduce the safety concerns linking gelatin to prion diseases when used as wall materials for microencapsulation	Dong and Hua *et al.*^131^
Whey protein isolate (WPI)–low methoxyl pectin (LMP)	Soybean oil	WPI-LMP particles formed through complex coacervation in a 1 : 2 ratio, due to electrostatic attractions. Stabilised HIPEs up to oil fraction of 79.02%, best at pH 3.5. Presence of NaCl, pH higher than 4 and freezing/thawing weakened the emulsification properties	• WPI is a common by-product of dairy industry that is readily available and has high nutritional benefits	Zhu *et al.*^102^
Zein with	• Zein is an environmentally friendly by-product of cereal processing that is easily modified			
	• Zein can easily form nanoparticles *via* self-assembly through the anti-solvent method, however it is too hydrophobic to stabilise Pickering emulsions alone			
– Corn fiber gum (CFG) (hemicellulose)	Medium chain triglyceride oil	Zein, modified with CFG to form complex particles using electrostatic attractions, improved the wettability and reduced the three-phase contact angle closer to 90° at a zein to CFG ratio of 2 : 1. The complex particle also gave better stability against flocculation and creaming than zein alone	• CFG is a waste product of corn fiber so cheaper than alternative polysaccharides (*e.g.* gum arabic)	Zhu *et al.*^120^
			• Modification with a small amount of CFG, gave much smaller particles that pure zein nanoparticles	
– Gum arabic (GA)	Thymol in soybean oil	Zein/gum arabic (GA) nanoparticles (ZGPs) fabricated through electrostatic interactions to stabilise Pickering emulsions with oil volume fractions up to *φ* = 0.6 with reduced agglomeration and lower interfacial tensions compared to pure zein nanoparticles. Used to encapsulate thymol, a microbial agent with poor water solubility, allowing for controlled release due to the interfacial ZGP layer	• Thymol loaded Pickering emulsions inhibited growth of *E. coli*	Li *et al.*^122^
			• GA has a good emulsion ability over a wide range of conditions *e.g.* pH, high temperatures *etc.*	
			• Long stability against coalescence (60 days)	
– Low acyl gellan gum (GG)	Corn germ oil	Presence of GG in the zein particles provided an emulsion gel where characteristics and wettability of particles were significantly affected by GG concentration. Smallest particles and droplets occurred at pH 4.2, with the emulsions being sensitive to pHs above and below this point	• GG is already an established emulsifier and stabiliser in food products	Jiang *et al.*^132^
– Starch	Soybean oil	Nanocomposites formed by adding varying concentrations zein to starch granules to modulate hydrophilicity of starch. Optimal particles formed at 1 : 0.3 starch : zein at pH 10 through a reverse dropwise method (starch dropped into zein). The contact angle of starch (54.9°) significantly increased upon complexation with zein (83.0°) and the approach to 90° suggests improved particle wettability. Emulsions had smallest and most uniform droplet sizes at pH 6 with 1% starch concentration	• Nanoprecipitation an environmentally friendly and easy preparation method	Li *et al.*^133^
			• Complexation with zein gave emulsions pH responsive abilities	
– Pectin	Sunflower oil	The effect of the degree of pectin methylation on hybrid pectin/zein dispersions (PZDs) and their ability to stabilise Pickering emulsions was examined. As the degree of methylation increased, the size of the PZDs formed decreased to smaller than zein nanoparticles alone and caused significant morphology change. The polydispersity and zeta potentials also decreased with increasing methylation. When methylation was above 35%, the PZDs had neutral wettability and low surface tension	• Well established method	Zhang *et al.*^134^
			• With methylation >35%, high oil fractions (*φ* = 0.6) could be stabilised against pH between 3-9 and presence of Ca^2+^	
			• Long term storage stability (30 days)	
– Pectin	Corn oil	Pickering HIPEs with an ordered 3D network were formed with an increasing pectin: zein ratio or at an ideal low pH (3.8) with the three-phase contact angle showing an improved partial wettability for all hybrid particles in comparison to pure zein. These HIPEs were able to encapsulate curcumin and protected it against UV radiation degradation	• HIPEs can be an alternative to partially hydrogenated oils in food products, lowering trans fats	Zhou *et al.*^135^
			• Pectin has beneficial gelling and thickening properties	

## 6. Summary

Pickering emulsions stabilised by various biodegradable solid particles and macromolecules have proven to be an excellent alternative to synthetic surfactant-based emulsions. The stability of Pickering emulsions investigated so far depends largely on the inherent properties of the stabilising material. Therefore, it is apparent that some challenges still remain, the most prominent of which is the poor stabilisation ability of unmodified bio-derived particles. Additional modification/functionalisation is then required, which can involve corrosive or toxic synthetic chemicals to impart desirable wetting properties for stable emulsion formation. However, the most recent work demonstrates that by combining natural materials, the ability to design bio-based particles with lower environmental impact is being dramatically improved. In addition, relatively high energy methods such as homogenisation and ultrasonication are often required to effectively absorb these solid particles at the oil–water interface. Therefore, future research warrants still more focus on probing lower energy routes to effective emulsification as well as on sustainable and greener approaches for functionalising these solid stabilisers, building on the active research in composite particles, to allow production of sustainable Pickering emulsions for safer consumer goods, biomedical formulations and industrial applications.

## Conflicts of interest

The authors declare no conflict of interest.

## Supplementary Material
